# The effect of animal-assisted interventions on the course of neurological diseases: a systematic review

**DOI:** 10.1186/s13643-023-02387-y

**Published:** 2023-11-25

**Authors:** Veronika Mittly, Cecilia Farkas-Kirov, Ágnes Zana, Kata Szabó, Veronika Ónodi-Szabó, György Purebl

**Affiliations:** 1 Institute of Behavioral Sciences, Semmelweis University, Nagyvárad Square 4, 1089 Budapest, Hungary; 2 South-Pest Central Hospital National Institute of Haematology and Infectology, Centre for Rehabilitation, Jahn Ferenc, Street 62-66, 1196 Budapest, Hungary; 3 Creanova Organisation and Management Development Consulting Ltd., Zichy Géza Street 5, 1146 Budapest, Hungary; 4 Támasz Foundation, Sándor Street 1, 7626 Pécs, Hungary; 5 Youthbuilders Foundation, Horánszky Street 26, 1085 Budapest, Hungary

**Keywords:** Rehabilitation, Nervous system diseases, Recovery of function, Animal-assisted therapy, Activities of daily living

## Abstract

**Background:**

In our experience, working with a therapy animal strengthens endurance, maintains motivation, provides a sense of achievement, and boosts overall mental resilience. The aims of this work were to summarize the results of quantitative research on the possibilities of animal-assisted intervention (AAI) among people with neurodegenerative and cerebrovascular diseases and to attempt to assess the effects of animal-assisted interventions in an objective manner and to find supporting evidence based on published literature.

**Methods:**

Our target groups are people diagnosed with Parkinson’s disease, multiple sclerosis, or stroke. A systematic search of relevant articles was conducted by two independent researchers in April 2021 and August 2023. The search for studies was conducted using PubMed, Google Scholar, Web of Science, Scopus, and Ovid databases, specifying keywords and search criteria. The qualitative evaluation of the research reports was conducted by four independent researchers, using the Newcastle–Ottawa Quality Assessment Form.

**Results:**

According to the scientific criteria and based on the Newcastle–Ottawa Quality Assessment Form, thirteen publications met the search criteria, out of which 9 publications were rated good and 4 publications were rated poor. Evaluating the publications we found evidence that AAI had a measurable impact on participants, as their physical and mental health status significantly improved; however, mental health improvement was more prominent.

**Conclusions:**

By developing evidence-based research methodology and standardized research settings, AAI could be measured effectively as part of health care practice. This would bring significant benefits to the rehabilitation of patients in need.

**Systematic review registration:**

PROSPERO CRD42021255776.

**Supplementary Information:**

The online version contains supplementary material available at 10.1186/s13643-023-02387-y.

## Background

The therapeutic use of animals goes back several decades. Animal-assisted interventions in healthcare institutions and among patients are widespread worldwide [1, 2]. Clinicians and rehabilitation professionals consider the involvement of therapy animals as an effective complementary therapy option, which can greatly assist doctors and therapists in encouraging patients to achieve therapeutic goals.

Approximately 40% of those in need of rehabilitation have a neurological problem underlying their functional impairment. According to a study, more than half of those affected by a cerebrovascular event develop speech and/or motor function impairment, and only about one third of them are able to resume their previous job. Due to the nervous system’s plasticity, the regular practice of specific tasks and daily skill development can help restore impaired function [3].

Besides stroke, the effects of animal-assisted interventions have also been examined in many studies in other areas of neurorehabilitation. Dog-assisted therapy carried out with Parkinson’s disease patients has been shown to have a positive effect on motor performance, mood, and quality of sleep [4]. The introduction of dog-assisted therapy for these patients was recommended by several experts to improve gait and balance and to treat depression, mood disorders, apathy, and anxiety often associated with the disease [5]. Equine-assisted/dog-assisted therapy has been successfully used with patients with multiple sclerosis. As a result of the rhythmic movement, the torsional and extension motions of the trunk musculature led to a significant reduction in lower limb spasticity and an improved sense of balance. In addition, psychosocial effects of the therapy have also been reported among these patients, who reported that their quality of life improved as a result of animal-assisted therapy [6, 7]. Hammer et al. found that animal-assisted therapy had a positive effect on the subjects’ gait, balance, coordination, and muscle strength [8]. The above is supported by another research study conducted among people with multiple sclerosis. According to this study, quality of life indicators significantly improved among people in the intervention group. Based on their scores on the Berg Balance Scale, their balance and coordination improved, and they became more confident in their movements [9].

Animal therapy can be implemented in several ways. Animal-assisted intervention is a goal-oriented activity for healing (AAT, animal-assisted therapy) and/or recreation (AAA, animal-assisted activity), which is carried out with animal involvement. In animal-assisted therapy, a trained therapy animal, a trained therapy animal handler, and a professional with expertise in the respective field who knows the individual or group work together to carry out a goal-oriented and personalized therapeutic intervention. In animal-assisted activity, however, only the beneficial effect of the presence of the therapeutic animal is utilized, primarily for recreational purposes and to improve quality of life [2].

Two issues definitely need to be clarified in connection with AAA and AAT. First, the question of efficacy, i.e., whether the positive clinical experiences that make AATs so popular can actually be measured in terms of clinical effectiveness (i.e., both symptom reduction and improvement in quality of life) and, if so, what the active impact factors of AATs might be. Based on several years of experience in animal-assisted intervention, our research team found animal-assisted intervention to be highly beneficial for the mental and physical well-being of both children and adults. The aim of the present systematic review is to summarize the results of quantitative research on the possibilities of animal-assisted activity or therapy among people with neurodegenerative and cerebrovascular disease. The rationale for this topic is demonstrated by the significant rates of functional impairment that develop and persist as a result of the abovementioned diseases. Our further aim is to provide a scientific approach to the effects of animal-assisted interventions, to objectify them, and—based on a summary of the reviewed studies—to identify evidence.

The target populations of the reviewed literature include people with Parkinson’s disease, multiple sclerosis, and stroke. The rehabilitation methods of these diseases share the common property that AAI is used as a complementary therapy, e.g., in addition to rhythm and music therapy or ergotherapy, and the aim is rehabilitation and improving the patients’ quality of life.

## Methods

We conducted our search on these three diseases. In the selected literature, only one publication mentions Parkinson’s disease patients and one mentions patients diagnosed with multiple sclerosis without quantifying them. The subjects of the other eleven studies were stroke patients. The literature predominantly presents AAI and related research on people with mental disorders; however, we decided not to include this in the search criteria, so that our focus would be more consistent and clear.

### Search strategy

Our review was previously registered at PROSPERO (registration number: CRD42021255776). A systematic search for relevant articles was performed by two independent researchers on 21 April 2021, on 29 April 2021, and was repeated on 29 August 2023. To obtain greater coverage of possible articles for our study we executed the search in multiple databases. Besides Pubmed, we searched an additional four databases: Google Scholar, Web of Science, Ovid, and Scopus. Based on relevant studies in this field, this combination should cover more than 90% of all relevant references [10, 11]. These studies were identified using the following terms and boolean operators: ("animal assisted therapy" OR "animal assisted intervention" OR "animal assisted activity" OR "pet therapy" OR "dog assisted" OR "canine assisted" OR "equine assisted" OR hippotherapy) AND (stroke OR "multiple sclerosis" OR Parkinson OR neurodegenerative OR neuromusculoskeletal). The range of year of publication was between 2001 and 2023.

### Selecting studies, inclusion, and exclusion criteria

To ensure that selected articles met all the inclusion criteria, title, abstract, and full text of records identified from the search were independently screened for eligibility by two researchers. The following criteria were applied for inclusion in our systematic review:

- Studies conducted on animal-assisted intervention
- Involving adult patients suffering from stroke, Parkinson’s disease, or multiple sclerosis

**Fig. 1:**
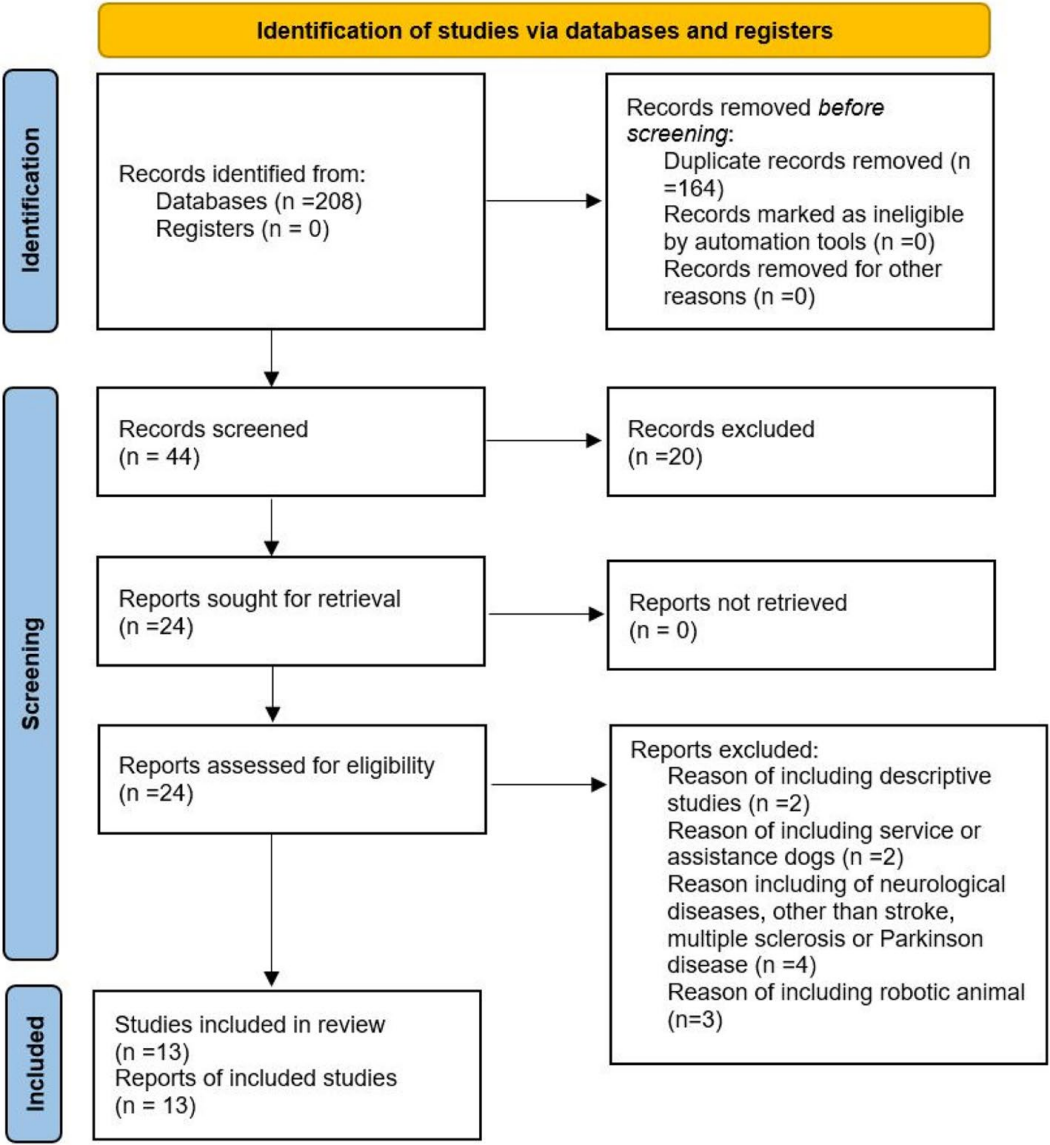
PRISMA chart of the study’s identification and selection process

In addition to randomized, controlled trials, we also included non-randomized, non-controlled studies, as only a few randomized controlled trials had been conducted in this field and we sought to present as many study designs as possible. Reports on children, publications on assistance or service dogs, case reports, systematic reviews and meta-analyses, and studies conducted on robotic animals were excluded. Discrepancies were resolved through discussion between the two review authors or by seeking advice from a third author. Publications in English were included. Duplicate records in the search results were removed using EndNote Web. Figure 1 shows the PRISMA flow chart of our study’s identification and selection process. An additional file shows the PRISMA checklist of our systematic review (see Additional file 1).

### Data extraction

Data extraction from all included studies was completed by four independent authors. The following information was extracted from each eligible article: the first author’s name, year of publication, sample size, targeted diseases, measurement tools and methods (e.g. questionnaires), and follow-up results.

### Quality assessment

The quality of each study was evaluated by four independent authors with the Newcastle–Ottawa Quality Assessment Form. This scale is one of the most used methods for assessing the quality of non-randomized studies in systematic reviews and meta-analyses. Its widespread use has led to a shared understanding and acceptance of its criteria and scoring system among researchers and reviewers. The Newcastle–Ottawa Quality Assessment Form provides clear criteria for assessing study quality, making the assessment process more transparent. Being well-structured, the Newcastle–Ottawa Quality Assessment Form provides a well-based framework for evaluating multiple domains of study quality.

We assessed eight items, grouped into three categories:

- 1. Selection of the study groups
- 2. Comparability of the groups
- 3. Outcome of the study

Studies were graded as good quality if they met 6 to 8 criteria, fair quality if they met 5 to 7 criteria, and poor quality if they met 0 or 1 point in the selection category or 0 points in the comparability category or 0 or 1 point in the outcome category.

We decided not to execute a meta-analysis for the following reasons: Although statistical methods are available for quantifying the heterogeneity, there are many qualitative differences in the enrolled studies which limit the interpretation of any comparison among them, independently from any kind of formal quantification. Firstly, these are different neurodegenerative conditions with different pathomechanisms. Secondly, all investigated conditions have significant inherent clinical heterogeneity, characterized by different subtypes and many forms of clinical manifestations which also hinder any comparison. Thirdly, the enrolled studies were not homogenous according to severity, age of onset, symptom lengths, and level of disability. Fourthly, considering treatment, different clinical protocols were administered that cannot be standardized statistically.

All of the abovementioned factors hinder the interpretability of any kind of quantitative statistical approach; therefore, we decided to conduct a stand-alone systematic review without meta-analysis.

## Results

Details of the abovementioned 13 articles are presented in Tables 1 and 2.

**Table 1:** Basic information about the selected articles

First author/year	Title	Country	Journal	Type of study
Beinotti et al. 2010 [12]	Use of hippotherapy in gait training for hemiparetic post-stroke	Brasil	Arquivos de Neuro-Psiquiatria	Experimental study
Beinotti et al. 2013 [13]	Effects of horseback riding therapy on quality of life in patients post stroke	Brasil	Topics In Stroke Rehabilitation	Original research
Berardi et al. 2022 [14]	The Effectiveness of Equine Therapy Intervention on Activities of Daily Living, Quality of Life, Mood, Balance and Gait in Individuals with Parkinson’s Disease	Italy	Healthcare	Outcome research study
Bunketorp-Kall et al. 2020 [15]	Motor Function in the Late Phase After Stroke: Stroke Survivors' Perspective	Sweden	Annals of Rehabilitation Medicine	Descriptive correlational study
Bunketorp-Kall et al. 2019 [16]	Effects of horse-riding therapy and rhythm and music-based therapy on functional mobility in late phase after stroke	Sweden	NeuroRehabilitaion	Descriptive correlational study
Bunketorp-Kall et al. 2017 [17]	Long-Term Improvements After Multimodal Rehabilitation in Late Phase After Stroke: A Randomized Controlled Trial	Sweden	Stroke	Single-blind, 3-armed randomized controlled trial
Lee et al., 2014 [18]	Effects of hippotherapy on recovery of gait and balance ability in patients with stroke	South-Korea	The Journal of Physical Therapy Science	Original research
Macauley, 2006 [19]	Animal-assisted therapy for persons with aphasia: A pilot study	USA	Journal of Rehabilitation Research & Development	Pilot study
Machová et al. 2019 [20]	The Effect of Animal-Assisted Therapy on the State of Patients' Health After a Stroke: A Pilot Study	Czech Rep	International Journal of Environmental Research and Public Health	Pilot study
Peppe et al. 2017 [21]	Targeting gait and life quality in persons with Parkinson's disease: Potential benefits of Equine-Assisted Interventions	Italy	Parkinsonism and Related Disorders	Pilot study
Rondeau et al. 2010 [22]	Effectiveness of a rehabilitation dog in fostering gait retraining for adults with a recent stroke: a multiple single-case study	Canada	NeuroRehabilitation	Multiple single case study
Sunwoo et al. 2012 [23]	Hippotherapy in adult patients with chronic brain disorders: a pilot study	South-Korea	Annals of Rehabilitation Medicine	Pilot study
Pálsdóttir et al. 2020 [24]	Equine-Assisted Intervention to Improve Perceived Value of Everyday Occupations and Quality of Life in People with Lifelong Neurological Disorders: A Prospective Controlled Study	Sweden	International Journal of Environmental Research and Public Health	Prospective controlled study

**Table 2:** Main original results of each study

Study	Duration (week)	Occasion/week	Lenght (minutes)	N_AAI	N_OAT	N_PC	Outcome	Mean (± SD)_AAI_baseline	Mean (± SD)_OAT_baseline	Mean (± SD)_PC_baseline	Mean (± SD)_AAI_follow up	Mean (± SD)_OAT_follow up	Mean (± SD)_PC_follow up
Beinotti et al. 2010 [12]	16	1	no data	10	-	10	Fugl-Meyer balance^a^	14,7 (3,8)	-	13,1 (6,2)	18,5 (3,6)	-	13,1 (7,3)
							Fugl-Meyer lower limbs	11,4 (1,6)		11,1 (0,9)	11,6 (1,3)		11,2 (1,8)
							BBS^a^	46,1 (12,9)		44,3 (12,3)	49 (13)		45,1 (14,2)
							FAC'	3,06 (0,8)		3,2 (1,0)	3,8 (0,9)		3,4 (1)
							gait cadence	96,1 (24,7)		96,5 (25,8)	92,5 (21)		100,9 (17,9)
							gait speed	0,7 (0,2)		0,8 (0,4)	0,8 (0,2)		0,8 (0,3)
Beinotti et al. 2013 [13]	16	1	30	12	-	12	MOSF36 SF-functional capacity^a^	40,5 (15,7)	-	50 (19,7)	51,5 (14,3)	-	40 (26)
							MOSF36 SF-physical aspects^a^	57,5 (35,5)		70 (28,4)	100 (0)		75 (35,4)
							MOSF36 SF-pain	97,5 (7,9)		63,9 (30,8)	91,9 (18,5)		70,6 (27,3)
							MOSF36 SF-general health state	75,3 (17,8)		75 (24,4)	85,9 (15,5)		77,7 (20,9)
							MOSF36 SF-vitality	63 (10,1)		57,5 (24)	77,5 (18,1)		61 (22,7)
							MOSF36 SF-social aspects	81,3 (19,3)		48,8 (28,5)	90 (12,9)		58,8 (36,8)
							MOSF36 SF-emotional aspects	83,3 (32,4)		50 (36)	96,7 (10,5)		70 (39,9)
							MOSF36 SF-mental health^a^	73,2 (22,5)		72,4 (13,7)	83,2 (16,9)		68,8 (18,5)
Berardi et al. 2022 [14]	5	2	45	17	-	-	SDS'	42 (7,45)	-	-	34 (6,84)	-	-
							UPDRS'	16 (8,45)			11 (7,43)		
							RAS'	30 (6,12)			38 (4,05)		
							Tinetti (total)'	16 (4,70)			20 (4,82)		
							PDQ 39 (total)'	70 (29,09)			52 (19,77)		
Bunketorp-Kall et al. 2020 [15]	12	2	no data	43	-	-	M-MAS UAS	50,33 (6,1)	-	-	no data	-	-
							BBS	50,4 (5,7)					
							TUG	14,3 (8)					
							Timed 10 m-WT-self selected'	12,7 (6,1)					
							Timed 10 m-WT-fast'	9,2 (4,8)					
Bunketorp-Kall et al. 2019 [16]	12	2	no data	41	41	41	10 m-WT Time: self selected^a^	15,05	10,03	13,37	12,83	9,49	13,74
							10 m-WT Time: fast^a^	9,71	7,26	9,84	8,52	7,12	10,72
							6MWT	361,29	437,69	374,89	388,35	453,38	383,49
							M-MAS UAS	49,63	51,39	50,8	50,76	52,01	50,98
Bunketorp-Kall et al. 2017 [17]	12	2	no data	41	41	41	SIS^a^	exact baseline data are not available			exact follow up data are not available, within group changes from baseline to follow up are publicated		
							TUG^a^'						
							BBS^a^'						
							BDL-BS						
							Grip strength (mean, L)						
							Grip strength (mean, R)						
							BNIS						
							LNS						
Lee et al., 2014 [18]	8	3	30	15	15	-	BBS'	40,4 (1,5)	40,5 (1,5)	-	42,7 (3,2)	41,7 (5,1)	-
							gait velocity'	38,3 (0,4)	38,4 (0,4)		39,6 (0,8)	38,8 (0,8)	
							step length asymmetry ratio'	0,32 (0,05)	0,32 (0,04)		0,19 (0,05)	0,29 (0,06)	
Macauley, 2006 [19]	12	1	30	3	3	-	WAB	74,37	74,37	-	75,4	73,6	-
							CSQ	-	-	-	8,8	7,5	-
Machová et al. 2019 [20]	6	2	20	6	-	9	Likert scale for mood	1	exact baseline data are not available	1	3,5	exact follow up data are not available	1
							diastolic blood pressure	72,5	exact baseline data are not available	77	75	exact follow up data are not available	72
							systolic blood pressure	exact baseline data are not available			exact follow up data are not available		
							heart rate	83	exact baseline data are not available	71	76	exact follow up data are not available	68
							Barthel index^a^	27	exact baseline data are not available	45	45	exact follow up data are not available	41
Peppe et al. 2017 [21]	5	1	60	3	-	-	GA Gait Velocity (m/s)	0,83	-	-	0,81	-	-
							GA Stride Length (m)	0,95	-	-	1,59	-	-
							GA Stance %	70,2	-	-	65,1	-	-
							GA Double Stance %	19,7	-	-	13,8	-	-
							UPDRS part III	19,6	-	-	13	-	-
							Attention and Executive functioning:Stroop Test Resistance to interference-Accuracy	19,5	-	-	26,5	-	-
							Attention and Executive functioning: Stroop Test Resistance to interference-Response Time	151,5	-	-	139,5	-	-
							Attention and Executive functioning: Trail Making Test-Subtest A	57,3	-	-	57,6	-	-
							Attention and Executive functioning:Trail Making Test-Subtest B	161	-	-	150,6	-	-
							STAI-Y1	30,3	-	-	30,3	-	-
							STAI-Y2	49,6	-	-	38,6	-	-
							GDS	9,3	-	-	5	-	-
							AES	32,6	-	-	29	-	-
Rondeau et al. 2010 [22]	3	4	60	4	4	-	10 m-WS^a^	28,75	-	-	15,37	-	-
							GA minor deviation	17,5	-	-	6,25	-	-
							GA major deviation	15,87	-	-	6,5	-	-
Sunwoo et al. 2012 [23]	8	2	30	8	8	-	K-BBS'	38,9 (11,9)	38,9 (13,1)	-	42,0 (12,0)	38,9 (11,9)	-
							POMA'	20,1 (5,3)	19,8 (5,7)		22,4 (5,5)	20,1 (5,3)	
							10 m-WT'	61,1 (56,0)	56,9 (50,1)		47,8 (44,0)	61,1 (56,0)	
							FAC	4,3 (0,7)	4,3 (0,7)		4,3 (0,7)	4,3 (0,7)	
							K-BDI	7,8 (8,1)	9,5 (9,8)		7,0 (8,9)	7,8 (8,1)	
							Ham-D	3,9 (3,6)	4,4 (4,1)		3,1 (3,9)	3,9 (3,6)	
							K-MBI	83,1 (8,1)	83,1 (7,6)		84,1 (8,5)	83,1 (8,1)	
Pálsdóttir et al. 2020 [24]	52	1	90	14	98	29	OVal-pd concrete^a^	2,5	-	2,6	2,8	-	2,6
							OVal-pd symbolic	2,3	-	2,4	2,6	-	2,4
							OVal-pd self rewarding	2,3	-	2,4	2,7	-	2,4
							SMBQ listlessness	5	-	4,5	4,7	-	4,5
							SMBQ tension	4,4	-	3,2	4	-	3,1
							SMBQ emotional and physical exhaustion	4,5	-	3,8	4,2	-	3,9
							SMBQ cognitive weariness	3,7	-	3,7	4,1	-	3,5
							EQ-VAS^a^	55	-	62	65	-	62

^a^Significant difference between intervention and control group, 'Significant difference within the intervention group, *p* < 0.05; *OAT* other additional therapy, *PC* Passive control

The main results of the study and results of our quality assessment are summarized in Table 3.

**Table 3:** Main results of the study and results of our quality assessment

First author/year	Targeted disease	AAI intervention type	Results	Newcastle–Ottawa score
Beinotti et al. 2010 [12]	Stroke	Equine-assisted therapy	↑ improvement in symptoms of motor impairment in the lower limbs*,↑ improvement on balance subscale*, ↑ improvement in the total study subjects *, ↑ improvement in Functional Ambulation Scale values, ↑ improvement in average cadence, ↑ improvement in average speed	3–1-2 (good)
Beinotti et al. 2013 [13]	Stroke	Equine-assisted therapy	↑ general health, ↑ functional capacity*, ↑ physical aspect*, ↑ mental health*, ↑ vitality, ↓ pain, ↑ emotional aspects	3–1-3 (good)
Berardi et al. 2022 [14]	Parkinson’s disease	Equine-asssited therapy	↑ mood*, ↑ mobility*, ↑ emotional well-being*, ↑ cognitive abilities*, ↑ social support, ↑ communication, ↓ physical discomfort, ↑ balance*, ↑ motor activity*	1–0-2 (poor)
Bunketorp-Kall et al. 2020 [15]	Stroke	Equine-assisted therapy	↑ all motor functions*; ↑gait speed*; ↑ balance, ↑ perceived stroke recovery	3–1-2 (good)
Bunketorp-Kall et al. 2019 [16]	stroke	Equine-assisted therapy	10 mWT ↑ self-selected*, ↑ fast speed*	3–1-3 (good)
Bunketorp-Kall et al. 2017 [17]	stroke	Equine-assisted therapy	↑ stroke recovery*, ↑ balance*, ↑ basic functional mobility*	3–1-3 (good)
Lee et al., 2014 [18]	Stroke	Equine-assisted therapy	↑ balance*, ↑ gait velocity*, ↓ step length asymmetry ratio*	3–1-2 (good)
Macauley, 2006 [19]	Stroke	Dog-assisted therapy	↑ communication, ↑ enjoyment, ↑ motivation	3–0-3 (poor)
Machová et al. 2019 [20]	Stroke	Dog-assisted therapy	– heart rate, ↑ diastolic blood pressure,—no significant change in systolic blood pressure, ↑ self sufficiency*, ↑ mood, ↑ well-being*	3–1-2 (good)
Peppe et al. 2017 [21]	Parkinson’s disease	Equine-assisted therapy	↑ motor skills; ↑gait variables; ↓apathy levels; ↓anxiety levels; ↓depression	2–0-3 (poor)
Rondeau et al. 2010 [22]	Stroke	Dog-assisted therapy	↑ walkig speed*; ↑ gait pattern	2–0-3 (poor)
Sunwoo et al. 2012 [23]	Stroke, traumatic brain disorder, cerebral palsy	Equine-assisted therapy	↑ balance*; ↑ gait funcion*; ↑emotion, ↑ activity of dailiy living	2–0-3 (poor)
Pálsdóttir et al. 2020 [24]	Multiple sclerosis, stroke, muscular disease, polyneuropathy, fibromyalgia, cerebral palsy	Equine-assisted therapy + activities in the care of the horse	↑ developing previous skills/learning new ones*, ↑ self esteem*, ↑ desire, will, zeal and motivation; ↓ perceived stress, emotional and physical exhaustion, tension, stress; ↑self estimated health*, ↑perceived health status*: ↑ balance, strength, energy, quality of sleep, well-being, confidence, self-confidence, mood, ↑ interacting with others in a group, ↑ empowerment, ↑ awareness	3–1-3 (good)

^*^Significant change in the intervention group

Table 4 summarizes the outcomes and their abbreviations used in the research.

**Table 4:** Outcome measures and abbreviations

Outcome measures	Abbreviation
Functional Ambulation Category Scale	FAC
Fugl-Meyer Scale	
Berg Balance Scale	BBS
NIH Stroke Scale/Score	NIHSS
Medical Outcomes Study 36-item Short-Form	MOSF36 SF
Stroke Impact Scale	SIS
Time up to go	TUG
Timed 10-Meter Walk Test	Timed 10 m-WT
Modified Motor Assessment Scale (according to Uppsala University Hospital)	M-MAS (UAS)
Subjective assessment of mood with Likert Scale	
Barthel index	BI
BDL Balance Scale	BDL-BS
Grip strength	
Barrow Neurological Institute screen for higher cerebral functions	BNIS
Letter–Number Sequencing Test	LNS
Western Aphasia Battery	WAB
Client Satisfaction Questionnaire	CSQ
heart rate + blood pressure	
Gait analysis	GA
State-Trait Anxiety Inventory	STAI
Hoehn and Yahr’s scale	
Unified Parkinson’s Disease Rating Scale	UPDRS
Zung Self-Rating Depression Scale	SDS
Parkinson’s Disease Questionnaire-39	PDQ-39
Rivermead ADL scale	RAS
Apathy Evaluation Scale	AES
Geriatric Depression Scale	GDS
Korean Berg Balance Scale	K-BBS
Tinetti balance assessment	
Tinetti Performance-Oriented Mobility Assessment	POMA
Korean Beck Depression Inventory	K-BDI
Hamilton Depression Rating Scale	Ham-D
Korean Modified Barthel Index	K-MBI
Occupational Value Assessment	OVal-pd
Shirom-Melamed Burnout Questionnaire	SMBQ
EuroQol Visual Analog Scale	EQ-VAS/EuroQol-VAS

In their study, Bunketrop et al. (2017 and 2020) assessed whether multimodal equine-assisted interventions targeting functional deficits and behavioral limitations are effective and whether functional improvement is sustained in the late phase after stroke. According to the evaluation, the proportion of people who observed improvement was higher in the experimental groups, with higher scores in gait ability, balance, and working memory, respectively. The improvements were sustained at 3 months and 6 months, as well [17, 15].

A study by Beinotti et al. (2013) investigated the impact of equine-assisted therapy on the quality of life of stroke patients. The functional capacity, physical fitness, and mental health of the subjects significantly improved in each area of the SF-36 questionnaire, compared to the control group [13].

Beinotti et al. (2010) examined the impact of equine-assisted therapy on the re-learning of gait in hemiparetic stroke patients. Compared to the control group, equine-assisted therapy significantly improved lower limb motor function and balance in the experimental group [12].

In their study, Berardi et al. (2022) investigated the impact of equine-assisted therapy on activities of daily living, quality of life, mood, balance, and gait in patients with Parkinson’s disease. Equine-assisted therapy resulted in statistically significant improvements in the occupational performance, mood, quality of life, gait, and balance of the participants [14].

Bunketrop et al. (2019) examined whether therapeutic riding and rhythm and music therapy have an effect on functional mobility in the late phase of stroke. In the equine-assisted therapy group, immediate and sustained improvement was shown in short-term gait ability. The music therapy group showed measurable improvement only at the 6-month measurement. The study confirms the beneficial effects of equine-assisted therapy on gait and functional task performance in the late phase of post-stroke rehabilitation [16].

Sunwoo et al. (2012) analyzed the effects of therapeutic riding on motor skills in adults with brain disorders. The balance and gait of patients significantly improved as a result of equine-assisted therapy. The study indicates that equine-assisted therapy improves the balance and gait speed of people with chronic brain disorders [23].

Lee et al*.* (2014) investigated the effects of equine-assisted therapy on balance and gait among adult stroke patients. In the experimental group, significant improvement was found in balance, gait speed, and gait asymmetry, while in the control group, only gait asymmetry was significantly improved. The research indicates that by improving balance and increasing step length and gait speed, therapeutic riding may reduce the risk of falls overall [18].

Machová et al. (2019) examined the benefits of dog-assisted therapy as a complementary therapy in the rehabilitation of stroke survivors concerning physiological and psychological status. Compared with the baseline measurements, there was no significant decrease in heart rate and systolic blood pressure in either group at the end of therapy, but there was a significant improvement in the subjective well-being of patients who had received AAT [20].

In their research study, Peppe et al. (2017) analyzed whether the motor and non-motor symptoms in patients with Parkinson’s disease could be improved in the short term by using equine-assisted therapy. According to the results, all participants in the study showed improvement in motor skills; however, this was not significant. The step length increased, the stance and double stance percentages decreased, and their attention, balance, mood, and quality of life improved in their short-term analysis [21].

Macauley examined the effectiveness of dog-assisted speech therapy for patients with aphasia and its impact on patient motivation. According to the results, the patients felt that they had made more progress during the AAT sessions and had been more motivated to undergo therapy combined with AAT. The effectiveness of AAT was reflected in subjective measures; the patients perceived the therapy as more enjoyable, they were more motivated, showed a wider range of emotions, and became more open in their communication [19].

In their study, Rondeau et al. (2010) examined the impact of the therapy dog as a therapeutic method and as a walking aid in the rehabilitation of stroke patients. In the presence of a therapy dog, gait speed and gait pattern improved significantly in all cases. According to their results, practicing with a therapy dog improves these scores of patients significantly more than using other aids [22].

The study of Palsdottir et al. (2020) aimed to analyze the impact of equine-assisted therapy on the participants’ daily engagement and perceived health and to understand what the interventions might mean for the participants' daily lives. The scores of perceived health were higher among those receiving equine-assisted therapy [24].

## Discussion

In our study, we attempted to review the available methods for quantification of the effectiveness of AAI, based on published literature. Since AAI is a diverse field, both qualitative and quantitative research methodologies were used in the reviewed publications. Therefore, we considered it necessary to review both evidence-based studies and published methods aiming to measure the effectiveness of AAT.

### Main findings

To summarize the original findings of our review, although there are multiple research about AAT and many best practices were published, the quantitative comparison of best practices is not possible because of the lack of standardized methodology and lack of standardized research protocols. As the quality assessment scores of the reviewed studies show there are only a few appropriate research methods in this field. Our original findings suggest that AAT is an emerging adjunctive treatment in the rehabilitation of various disorders, which has gained significant popularity. Future studies with homogenized patients’ groups and standardized methodology are needed to increase the comparability of the studies and therefore provide more robust evidence of the efficacy of AAT. Thus, based on the results, we may conclude that AAI significantly improved both the physical and mental well-being of participants, but the mental improvement was more outstanding. According to the results, the physical rehabilitation achieved by AAI is slower, while psychological coping can be facilitated in the short term by the therapy animal.

### In-depth comparison of the reviewed research

Of all the research studies we reviewed, three studies examined the effects of animal-assisted intervention on participants based on how the intervention complementing the usual treatment impacted them (Beinotti 2010, 2013, Machová). In addition, two articles examined the effect of animal-assisted interventions carried out in addition to standard therapy, and the effects of other additional interventions besides standard therapy (Bukentrop 2017, 2019).

This is supported by Palsdottir’s research. In their study, participants spent 30 min with effective equine-assisted therapy once a week, complementing this by patients spending 60 min connecting with each other, attuning to the therapy animal, and preparing for the joint activity (social connection, building bridges). This is an important intervention from both the musculoskeletal and the mental perspectives. From Palsdottir’s research, it is clear that AAI induced significant positive changes in the patients’ mental state. Machová et al. indicated that those patients who received AAT felt better despite showing no significant changes in heart rate or blood pressure. The beneficial effect of AAT on patients' well-being is also demonstrated by the subjective mood ratings on a Likert scale that patients completed before and after the session. By improving the mood of the patients, AAT has an indirect impact on the successful rehabilitation of patients. It encourages proactivity in the treatments they receive, improves their interaction with other therapists, and thus improves their relationship with the whole team providing the treatment (Machová, 2019).

In the studies we reviewed, the use of horse-assisted interventions was over-represented compared to dog-assisted ones; however, the results of the research studies suggest that horses are more likely to assist with physical development, while dogs are more likely to assist with mental and social development. Beinotti, Bunketorp, Lee, and Sunwoo et al. supported the beneficial effects of equine-assisted therapy on balance, gait function, and hand and arm use. However, Peppe’s small sample size study found that a 5-week course of equine-assisted therapy in Parkinson’s patients improved motor skills and also reduced anxiety and apathy among participants. The developmental impact of a dog-assisted intervention is supported by the results of Machova and Macaulay. They found that dog therapy increased motivation, mood, and self-reliance in stroke survivors.

### Limitations

Clarity is made difficult by the fact that concepts of AAT are still not applied uniformly, as experts do not refer to exactly the same things by them. Despite this incongruity, we tried to use the terminology related to animal-assisted intervention consistently. Thus, we used the term “dog-assisted therapy” for interventions with dogs and “equine-assisted therapy” for interventions with horses, but the activities carried out in the studies with dogs and horses differ from one study to another.

Another important limitation is the diversity of measurement tools. Physical (gross motor function, fine motor function, and balance) and mental changes (mood, motivation, and well-being) are measured very differently, making it difficult to compare results. From the publications we analyzed, we have identified approximately thirty different scales, such as FAC, Fugl-Meyer Scale, BBS, NIHSS SIS, TUG, Timed 10 m-WT, M-MAS (UAS) for the measurement of physical changes, and STAI, OVal-pd, EQ-VAS/EuroQol-VAS SMBQ for the measurement of mental changes. There are also tools that measure both physical and mental states, such as the MOSF36 SF. There are also measurement tools that we identified in several publications, e.g., TUG, Barthel index, and Timed 10 m-WT.

Also, an important limitation is the disproportionality of studies with different species. In the literature reviewed, equine-assisted therapy is over-represented (3 therapies with dogs, 10 therapies with horses), but we have no information on the number of AAI in the world and the proportion of dog-assisted therapies and equine-assisted therapies, and on how many of these attempts to measure the impact of animal therapy. Our research team could analyze only published papers. It is a well-known fact that in past centuries, in the case of dogs and horses (as opposed to other animals), cooperation with humans was a main selection criterion during the course of breeding, and both species have a long tradition of being involved in tasks and being specifically trained.

The quality of the published research varies widely. In the present review article, we examined thirteen publications with the following results: 9 publications were rated good, and 4 publications were rated poor. One possible reason for this may be the scarcity of available literature on the topic that meets the criteria and the novelty of the research field. Due to the small number of papers published on this topic and meeting our quality criteria, we did not limit the scope of the present review to one group of animals, one disease, or one type of animal-assisted intervention. The low number of quality articles published posed a challenge to our research. On one hand, this paints an incomplete/distorted picture of this form of complementary rehabilitation; on the other hand, it also draws attention to the fact that there have been very few studies of appropriate excellence on this topic. Since this field has been undergoing explosive development, our study calls attention to the fact that many more qualitative research studies are necessary to provide an overview of the subject. Besides the low number of quality publications, the other major limitation was methodological heterogeneity. Although the definitions are provided and there is literature that specifies criteria, e.g., for animal-assisted therapy, on reviewing the research reports we found that these definitions are not used consistently in practice. Furthermore, we could find no information on whether there are comparable forms of quality control measurement in place in different countries, and due to their absence, the use of measurement tools and interventions may differ from one country to another, or even from one research study to another. The length of musculoskeletal and social interventions varies. The interventions range from a few weeks or months to complex programs lasting a year. This makes it difficult to compare the effectiveness of AAIs of varying lengths.

In most studies, AAT was used as an adjuvant to conventional medical therapy, but the studies do not provide clear-cut information on whether patients with similar illness severity were selected in the experimental and control groups. Therefore, as mentioned earlier, quantitative comparison of efficacy has major limitations. Although Parkison’s disease seems to be associated with a higher efficacy of AAT than MS, we cannot draw definite conclusions due to significant confounders such as the great variability of the severity, symptoms, and course of the conditions. Affected brain areas can be very different even in the case of one particular disease, which may have direct effects on psychomotor functions. There are many psychological confounding factors (mood, apathy, level of motivation, locus of contol, etc.) which also affect the response to any intervention. Another important factor is the great variability of the studies and the lack of standardized methodology for measuring efficacy. Future studies with standardized assessment and methodology and more homogenous participants groups considering psychomotor functions, disease severity, and mental health state can be eligible for quantifying efficacy differences.

### Strengths

As interest in AAT has grown rapidly and the number of scientific publications on the subject has increased significantly over the last 15 years, there has been an increasing need for both providers and users to review the state of the art in AAT. An important strength of the paper is that it attempts to investigate the effectiveness of AAT by summarizing highly heterogeneous studies that differ in methodology, quality, and target group. The publication following the 2006 peer-reviewed paper, which met the selection criteria in terms of quality, is from 2010, followed by papers published in 2013 and 2014. The fact that 7 of the publications we analyzed were written in the past 7 years suggests that interest in the topic has increased in recent years, with more and more people using AAI. The number of research studies on AAI is growing, and its literature is expanding at a faster pace. A further strength of our paper is that the approach presented in the review provides a quality framework for the development of methodological features for future research. If the presented framework is followed in future studies, comparability, and evidence-gathering would be much easier.

### Implication for practice and research

There are few evidence-based studies available, and their summary is insufficient to draw any far-reaching conclusions; however, we may venture to conclude that AAT is an effective adjuvant treatment for the studied diseases, adding significant value to conventional therapies, particularly for psychiatric symptoms and quality of life, and, in the case of equine-assisted therapy, for movement rehabilitation. Therefore, we need to emphasize the importance of developing a uniform measuring methodology, a larger sample size, and detecting significant changes. In the case of control group studies, it would be important to use simultaneous complementary therapies, such as dog-assisted therapy and art therapy in the two analyzed groups. By developing evidence-based research methodology and standardizing the research setting, the effects of animal-assisted therapy could be effectively measured as part of healthcare practice, which would bring about significant benefits in the rehabilitation of patients in need. Interventions of different lengths could be subject to further research in order to identify the length of intervention that is the most effective, and the systemic change a 12-month intervention can trigger in patients’ lives.

It would be a step forward in quantifiable interventions with measurable impact if professionals used the definitions uniformly. It would also be important for research and interventions to have a regulated professional framework for animal-assisted therapy, within which the professionals have the flexibility to tailor the therapy to the clients’ needs.

In connection with the measurements, the idea may arise that control group studies conducted in health care institutions would be more fit for the purpose. However, given the heterogeneity of the patient group and the pharmacotherapy used, the implementation of this cannot be standardized as of yet. In contrast, biometric measurements could be helpful in measuring the impact of AAI.

We would like to confirm that although this is a difficult area to assess, at the same time, an evidence-based approach is indispensable. Psychometric tools (depression, anxiety) are key in measuring changes in mental health.

## Conclusion

Based on the results, we concluded that placing emphasis on strengthening coping skills and improving subjectively experienced quality of life and emotional states is important when using animal-assisted interventions. Therefore, AAI has an indirect positive effect on well-being, which is important not only for the individual, but for society as a whole. In our own experience, the effects of animal-assisted therapy are manifested sooner in psychological and mental factors than in physical fitness, and a few appropriate sessions can already have a positive effect on patients’ motivation, general well-being, and quality of life. However, our experience so far suggests that AAI has an effect on physical fitness as well, mainly in terms of reducing pain and increasing mobility.

Our studies have certainly shown that AAT is an area worth investigating, but one that really needs practice guidelines and standardized research methodology. As the popularity of AAT continues to grow among users, it is very important that providers not only respond to these needs, but do so in a way that is evidence-based and according to appropriate standards. The true place of AAT in patient care can only be accurately determined on the basis of methodological excellence both in research and clinical practice.

### Supplementary Information

**Additional file 1.** PRISMA checklist.

## Data Availability

All data generated or analyzed during this study are included in this published article.

## Abbreviations

AAA: Animal-assisted activity
AAI: Animal-assisted intervention
AAT: Animal-assisted therapy
AES: Apathy evaluation scale
BBS: Berg balance scale
BDL-BS: BDL balance scale
CSQ: Client satisfaction questionnaire
EQ-VAS: EuroQol visual analog scale
FAC: Functional ambulation category scale
GA: Gait analysis
GDS: Geriatric depression scale
Ham-D: Hamilton depression rating scale
K-BBS: Korean berg balance scale
K-BDI: Korean beck depression inventory
K-MBI: Korean modified barthel index
LNS: Letter–number sequencing test
M-MAS (UAS): Modified motor assessment scale (according to Uppsala University Hospital)
MOSF36 SF: Medical outcomes study 36-item short-form
NIHSS: National institute of health stroke score
OVal-pd: Occupational value assessment
POMA: Tinetti performance-oriented mobility assessment
SIS: Stroke impact scale
SMBQ: Shirom-melamed burnout questionnaire
STAI: State-trait anxiety inventory
Timed 10 m-WT: Timed 10 metre walk test
TUG: Time up and go
UPDRS III.: Unified parkinson’s disease rating scale III
WAB: Western aphasia battery
